# Tissue-engineered patient-derived osteosarcoma models dissecting tumour-bone interactions

**DOI:** 10.1007/s10555-024-10218-2

**Published:** 2024-11-27

**Authors:** Tina Frankenbach-Désor, Isabella Niesner, Parveen Ahmed, Hans Roland Dürr, Alexander Klein, Thomas Knösel, Jonathan Gospos, Jacqui A. McGovern, Dietmar W. Hutmacher, Boris M. Holzapfel, Susanne Mayer-Wagner

**Affiliations:** 1 Department of Orthopaedics and Trauma Surgery, Musculoskeletal University Center Munich (MUM), LMU University Hospital, LMU Munich, Marchioninistr. 15, 81377 Munich, Germany; 2 Department of Orthopaedics and Trauma Surgery, Orthopaedic Oncology, Musculoskeletal University Center Munich (MUM), LMU University Hospital, LMU Munich, Marchioninistr. 15, 81377 Munich, Germany; 3 Institute of Pathology, Ludwig-Maximilians-Universität (LMU) Munich, Thalkirchner Str. 36, 80337 Munich, Germany; 4 Centre for Biomedical Technologies, School of Medical, Mechanical and Process Engineering, Queensland University of Technology (QUT), 60 Musk Avenue, Kelvin Grove, QLD 4059 Australia; 5 Centre for Biomedical Technologies, School of Biomedical Sciences, Queensland University of Technology (QUT), Translational Research Institute, 37 Kent Street, Woolloongabba, QLD 4102 Australia; 6 Max Planck Queensland Center for the Materials Science of Extracellular Matrices, Queensland University of Technology (QUT), 2 George Street, Brisbane, QLD 4000 Australia

**Keywords:** Osteosarcoma, Patient-derived xenograft models, Bone tissue engineering, Patient-specific cancer models, Bone microenvironment, Bone malignancies

## Abstract

Osteosarcoma is the most common malignant bone tumor, primarily affecting children and young adults. For these young patients, the current treatment options for osteosarcoma impose considerable constraints on daily life with significant morbidity and a low survival rate. Despite ongoing research efforts, the 5-year survival rate of first-diagnosed patients without metastases has not changed in the past four decades. The demand for novel treatments is currently still unmet, in particular for effective second-line therapy. Therefore, there is an urgent need for advanced preclinical models and drug-testing platforms that take into account the complex disease characteristics, the high heterogeneity of the tumour and the interactions with the bone microenvironment. In this review, we provide a comprehensive overview about state-of-the-art tissue-engineered and patient-specific models for osteosarcoma. These sophisticated platforms for advanced therapy trials aim to improve treatment outcomes for future patients by modelling the patient’s disease state in a more accurate and complex way, thus improving the quality of preclinical research studies.

**Graphical Abstract:**

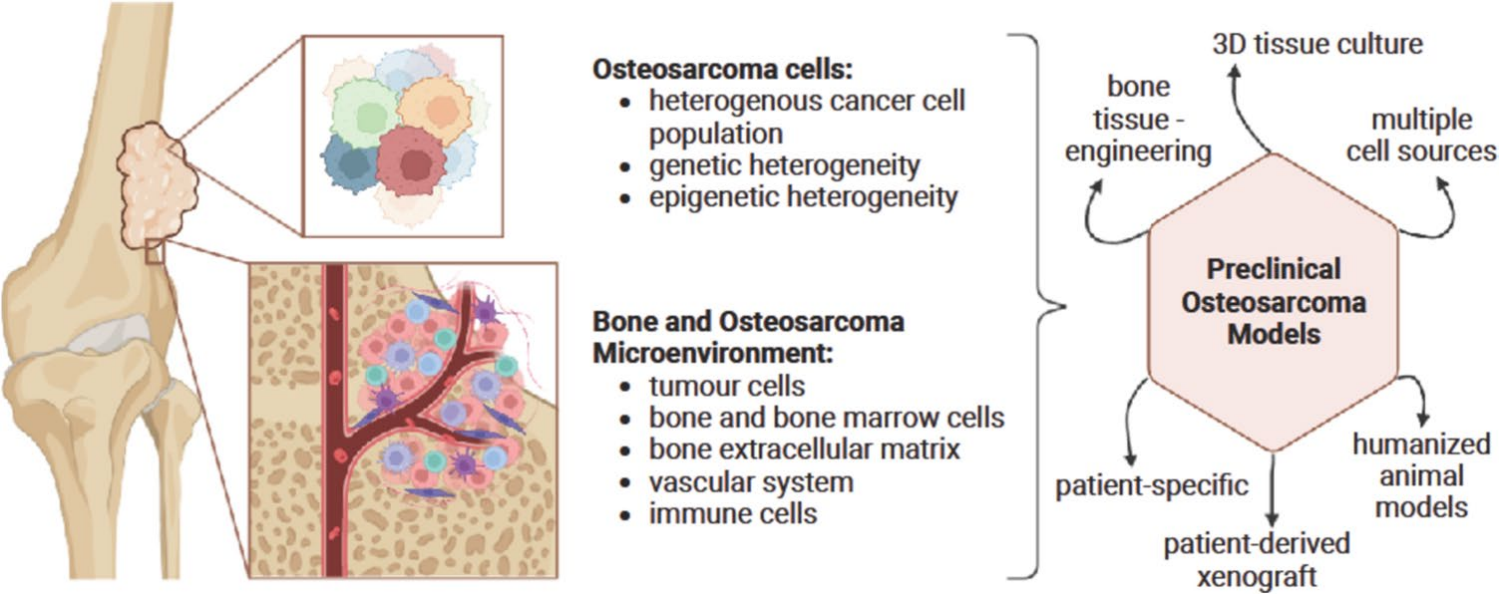

## Introduction

For years, a significant challenge in medical research has been the ability to translate preclinical research findings into clinically effective treatments for patients [1]. More than 80% of therapies found to be effective and safe in preclinical research fail in clinical testing phases [2]. Among these, new potential therapies fall short most often in oncology with musculoskeletal diseases following in third place [3].

The insufficient translatability of preclinical research findings into clinical settings is largely attributed to the utilization of inadequate model systems [2, 4]. To overcome this obstacle and to enhance the validity of preclinical outcomes, new models are being developed with the goal of better representing various disease states present within patients. There is an increasing shift towards complex 3D cell culture, patient-specific and/or humanized mouse models. Moreover, the role of the microenvironment in certain disease patterns is considered more carefully to increase model translatability [4–6].

The field of osteosarcoma research is no exception to the widely experienced translational gap. On the contrary, the translational gap might be even worse for osteosarcoma despite tremendous research efforts. The 5-year survival rate of patients with a first diagnosed localized tumour has not improved in more than 40 years, as treatment options essentially have not changed since the late 1970s [7]. About 30–40% of the patients will experience recurrent disease [8]. Due to the current lack of validated second-line treatment options, patients with progressive disease face poor survival rates. Metastasis to the lung happens frequently and is considered the most fatal complication [9, 10]. Thus, new treatment options are desperately needed, particularly for metastatic and relapsed disease.

In this review, first, a brief overview of the disease as well as its great complexity is given highlighting the various challenges of modelling osteosarcoma. Furthermore, we summarize the current state-of-the-art of complex microenvironment as well as patient-derived osteosarcoma models and evaluate their advantages and shortcomings in serving as drug testing platforms.

## Osteosarcoma

### Disease background

**Fig. 1:**
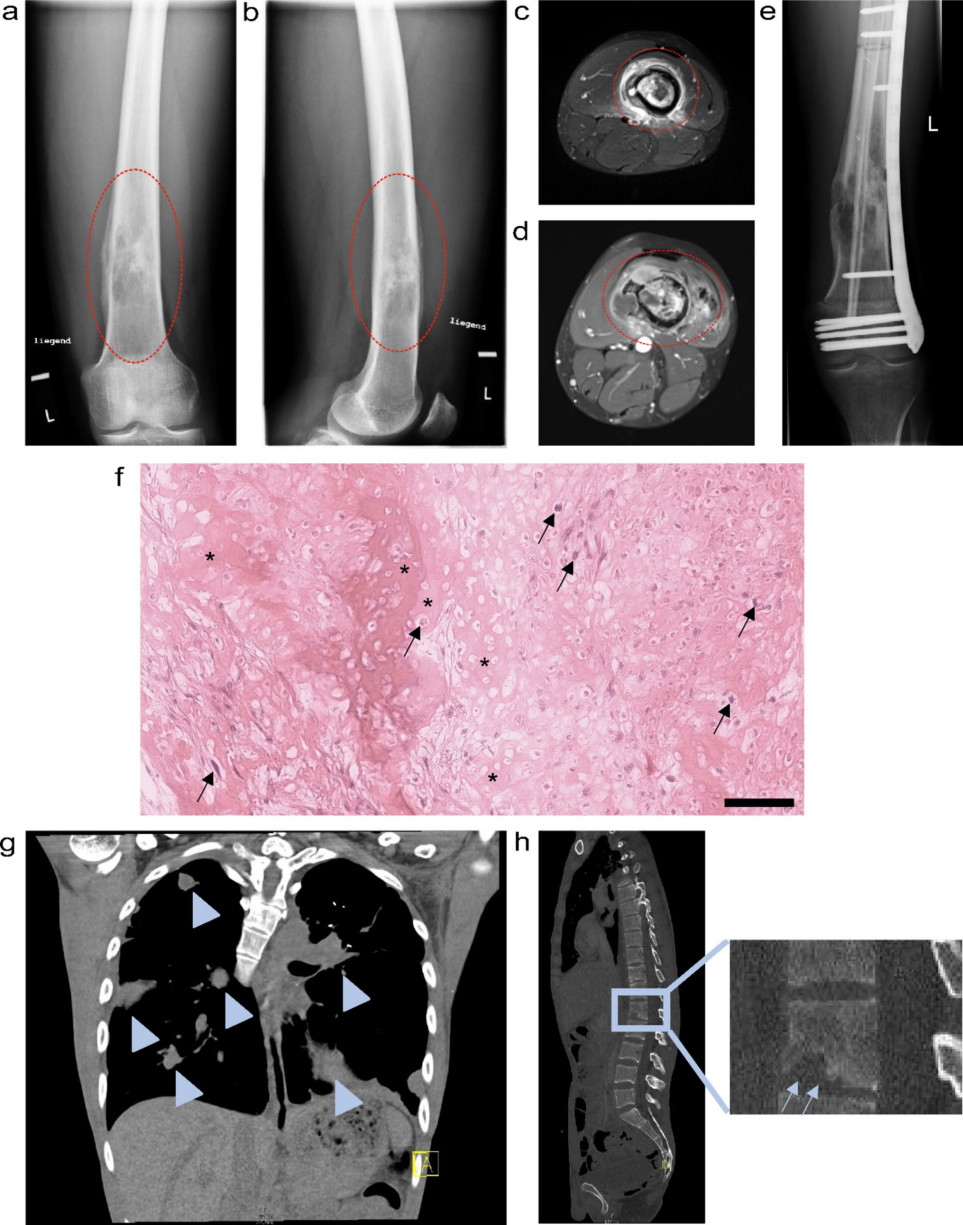
Clinical example case. ***a***–***c*** Male patient was first diagnosed with a high-grade osteosarcoma of the left femur at age 21. Radiographs and MRI image at the time of first diagnosis: anterior (***a***), posterior (***b***) and MRI transversal view (***c***). Osteosarcoma is visible in the diaphyseal region of the distal femur in the region indicated by the dotted red lines and was confirmed pathologically after a true cut biopsy was conducted. Neoadjuvant chemotherapy, with methotrexate, doxorubicin and cisplatin, according to the EURAMOS-1 protocol was initiated shortly after. ***d*** MRI showing condition before resection surgery, about 3 months after initial diagnosis: tumour progression in size along with extended infiltration of the bone marrow and the surrounding muscle area. ***e*** Postoperative radiograph shows the left femur after a wide tumour resection, intraoperative radiation sterilization of the specimen and orthotopic replantation. In addition, a non-vascularized fibula transplant and a plate osteosynthesis was used. Chemotherapy was continued post-surgery. ***f*** Haematoxylin & eosin staining of the resected osteosarcoma reveals highly atypical hyperchromatic spindle cells (indicated by black arrows) and atypical osteoid formation (indicated by asterisks): scale bar, 100 µm. ***g***, ***h*** Within the first year after resection surgery, relapse of the primary tumour was detected with metastatic disease in multiple locations: bipulmonary lung metastases (***g***, indicated by arrowheads) and bone metastases in the spine (***h***) were detectable, amongst others. Spinal metastasis leads to the partial destruction of the vertebrae, as indicated by the blue arrows in ***h***. All attempts of subsequent secondary therapy ultimately failed, the patient died after 1 year of relapsed disease management. The images were provided with courtesy of Prof. Dr. med. Hans Roland Dürr and Prof. Dr. med. Thomas Knösel

Osteosarcoma is the most common primary malignant bone tumour. With an incidence of approximately three cases per million people worldwide, it is considered a rare disease [11]. Osteosarcoma can occur in patients of all ages; however, mostly children and young adults are affected (see Fig. 1). A second peak in incidence occurs in adults of old age [12].

Osteosarcoma originates from malignant mesenchymal cells committed to osteoblastic differentiation [13]. Thus, the production of tumour osteoid matrix is characteristic of the disease. Most osteosarcomas occur near the metaphyseal growth plate of the long bones of the arm and legs, most often in the femur (30%), tibia (15%) and humerus (15%) [12].

The 5-year overall survival rate is more than 70% [14]. The current treatment standard for localized disease consists of neoadjuvant chemotherapy, followed by surgical resection of the tumour and adjuvant chemotherapy. Combination chemotherapy consisting of high-dose methotrexate, doxorubicin and cisplatin (MAP) is widely considered the gold standard treatment [7, 15]. Around 20% of patients with a first diagnosed osteosarcoma present with metastasis, most often to the lung [9]. However, the estimated occurrence of occult metastases in this patient group is expected to be much higher [16, 17]. Curative treatment approaches mandate complete resection of the primary tumour as well as the detected metastases [15]. Since the introduction of the MAP chemotherapy regimen in the late 1970s, no evidence-based significant improvements affecting patient survival could be achieved [7]. Moreover, there are few prospective randomized trials beyond first-line therapy. Thus, treatment guidelines are often unspecific or include off-label use of substances that were shown beneficial in smaller studies [15]. The lack of well-established second-line treatment options is a problem, as tumour recurrences happen as often as in 30–40% of patients with local disease [8]. Treatment of this substantial group of patients becomes more complicated as the disease progresses. Effective second-line treatment options as well as drugs specifically targeting lung metastases are desperately needed [9, 10]. The development of lung metastases is considered the most fatal complication of osteosarcoma. It is estimated that a potent drug being able to prevent lung metastasis could prevent around 70% of overall osteosarcoma-related deaths [10].

The clinical case in Fig. 1 shows an example of why novel therapies for advanced and metastatic disease patients are desperately needed. In the example, first-line treatment failed—progression of tumour size during neoadjuvant therapy (compare Fig. 1a–c to d) already indicates poor course of the disease. Resection surgery (Fig. 1e) is thus rapidly followed by disease recurrence as well as extensive metastatic spread (Fig. 1h and e) which, despite all efforts taken with currently available second-line therapies, is ultimately fatal for the patient.

### Osteosarcoma: a heterogeneous disease

Even though the patient’s age and histological subgrouping are identified and considered in clinical practice [18], osteosarcoma is a very heterogeneous disease beyond these factors.

Most tumours emerge gradually: one single mutation after the other, typically over the course of many years or decades, until a tumour is formed. Osteosarcoma, on the other hand, is known for a high rate of complex and rapid mutations across the whole genome; due to copy number alterations, kataegis and chromothripsis [19]. Localized hypermutations called kataegis occur in about half of all osteosarcoma patients [11, 20]. Chromothripsis describes a large number of genetic mutations happening at a single event, causing around 77% of genomic complexity [21, 22]. The p53 and rb1 mutations occur most frequently, however, as described above are complemented by a fast number of further (driver) mutations [10]. Moreover, extensive epigenetic modulations, although not fully understood yet, are believed to further contribute to the heterogeneity of the disease [23].

Briefly, osteosarcoma shows remarkable heterogeneity when it comes to genomic mutations. The described heterogeneity of the disease does not only present between individual patients but may also occur within a single patient during recurrence and metastasis [5]. Hence, possible interventions targeting specific mutations and the affected pathways may at times benefit only a small subset of the patient cohort. Therefore, patient-specific treatment approaches and clinical trials that take the complex genomic heterogeneity into account are needed to advance the current treatment standard [24]. This might be especially important for patients with a low response to MAP treatment, suffering from recurrence and metastatic disease [15]. As osteosarcoma is a rare disease, clinical trials thoroughly evaluating individual treatment for a subset of patients will present a challenge and therefore must be prefaced by meaningful preclinical data [7].

### Impact of the bone microenvironment on osteosarcoma growth

As shown above, osteosarcoma in itself is a highly complex disease. However, the complexity extends to the significant interaction of osteosarcoma cells with their surrounding microenvironment. The osteosarcoma tumour environment is also heterogeneous: In the context of the primary tumour, it includes the bone environment, including blood and immune cells, fibroblasts, endothelial cells, extracellular matrix (ECM), signalling molecules and extracellular vesicles [25]. With metastasis, the environment expands to the tissues of the metastatic site, such as the lung [19]. As further research is conducted to comprehend the intricacies of these interactions and their consequences on the disease, the relationship between osteosarcoma and the microenvironment becomes increasingly interesting for constructing models and ultimately, devising novel therapeutic approaches [19, 26].

Interaction with the bone itself is believed to be a crucial factor during all stages of tumorigenesis, although not entirely understood at present. Interactions with osteoclasts are evident, although at times contradictory and critically discussed. There seems to be a “vicious cycle” started by osteosarcoma cell secretion of cytokines that lead to osteoclast-stimulation or upregulation of receptor activator of nuclear factor-κB ligand (RANKL) in osteoblasts. Put simply, those interactions may ultimately lead to dysregulated bone lysis, which promotes tumour growth based on the released factors and fosters further destruction of the healthy bone [11, 27]. On the other hand, higher osteoclast activity levels were observed to prevent pulmonary metastasis. The authors hypothesize upregulation of osteoclastic activity initially created an incentive for the osteosarcoma cells to stay at their primary side due to an abundance of factors favouring tumour growth. However, as the disease progresses, further (epi)genetic changes might lead to inhibition of osteoclastic activity ultimately enabling metastasis [28, 29].

Numerous interactions with cells within the bone microenvironment have been described in the literature. These interactions occur through various signalling molecules present in the bone environment, as well as through extracellular membrane vesicles [27, 30].

It is also worth mentioning that the ECM plays a crucial role in osteosarcoma tumorigenesis, as summarized in Cui et al.’s recent detailed review [25]. Briefly, the authors present literature showing how different aspects of the ECM interact with the tumour cells and enable osteosarcoma progression and metastasis in particular. Different types of collagens, fibronectin, laminins and proteoglycans all play differing roles in influencing tumour cell growth, migration, adhesion, invasion and metastasis. Furthermore, some factors like collagen III and fibronectin are linked to chemoresistance [31, 32].

In summary, adequate modelling of the osteosarcoma microenvironment is complex, and different microenvironment systems may be of interest depending on the location of the tumour studied (and even vary for local vs. metastatic site). Furthermore, tumour-microenvironment interactions are not fully understood yet and are subject to continued research studies. Nevertheless, the presence of an intact microenvironment is crucial for successful osteosarcoma research and must be considered to produce meaningful preclinical data. Due to the vast complexity of osteosarcoma-microenvironment interactions, this review will focus specifically on the bone microenvironment.

## Modelling osteosarcoma

Osteosarcoma is a complex disease with diverse tissue composition and morphology. Therefore, creating an “ideal” model for osteosarcoma is impossible, as it would need to comprehensively replicate all facets of the disease, including those that are presently unknown, across the entire patient population. As a result, our efforts should focus on creating and working with a model systems technology platform. This platform should be built on several different models that, taken together, are able to replicate the current knowledge of the disease as closely as possible to the situation in actual patients and employ state-of-the-art methodologies. In the author’s opinion, what makes a model successful is its ability to describe biological processes that correspond to those in patients and thus have real-life usefulness. At the same time, a good model should be feasible for application in laboratories. As already stated, we consider two main factors necessary in a comprehensive model: tumour-microenvironment interactions and patient-specificity to account for the heterogeneity of the disease. Tumour-microenvironment interactions influence nearly all steps of tumour development, from treatment success to metastasis, and therefore should be included to enhance translatability. Furthermore, using (fresh) patient-derived tissue is crucial for maintaining the patient’s disease characteristics. However, even if researchers could faithfully model the entirety of one patient, this single patient’s disease would not universally reflect all osteosarcoma patients, due to high interpatient heterogeneity. Therefore, a universal approach will not suffice, and the aim should be a panel of patient-specific models. In this review, we will discuss human osteosarcoma models that include (1) the bone microenvironment and (2) patient-derived tissue. We will evaluate their potential for use as drug testing platforms and their ability to accurately represent human disease based on current knowledge.

### Modelling the human bone microenvironment for osteosarcoma research

The human bone environment plays a crucial role in osteosarcoma tumorigenesis and metastasis [25]. Therefore, a rising number of researchers also take the bone microenvironment into account when modelling osteosarcoma, exploring new therapeutic targets and testing their efficacy. In the following section, we present strategies that have recently been used to model the bone microenvironment in the context of osteosarcoma research in particular. Moreover, Table 1 provides an overview of microenvironment models used in recent studies.

**Table 1:** Selection of microenvironment models for primary bone malignancies of human origin

Category				Tumour cell source	Other tissue sources	Target niche	Literature
2D	*In vitro*	Transwell indirect co-culture		MG-63 and U2OS OS^1^ cell lines	hMSCs	Bone (marrow) microenvironment	[33]
		Direct co-culture		HOS, HG-63, SAOS-2 and U2OS OS cell lines	hMSCs		[34]
3D	*In vitro*	Spheroids			/	Tumour ECM	[35–37]
		Spheroids + 2D co-culture		MG-63 OS cell line (spheroids)	HUVECs (2D, vascular component)	Tumour ECM + vascular niche	[38]
		scaffolds	Silk sponge	SAOS-2 and U2OS OS cell lines	/	Tumour ECM	[39]
			MgHA/Coll + HA	MG-63 and U2OS OS cell lines	/	Bone ECM	[40]
			PCL	TC-71 ES^2^ cell line	hMSCs (decellularized)		[41]
			PU	SAOS-2 OS cell line	hMSCs (lysed)		[42]
			Sponge-like Col1/nHA	MG-63 OS cell line	/	Bone	[43]
			Decellularized bovine bone	RD-ES and ATCC ES cell lines (spheroids)	hMSCs		[44]
			Scaffold-gel hybrid	MG-63 and 143B OS cell line (tumouroids)	NuOSS™ cancellous bone granules	Bone (compartmentalized: bone and ECM)	[45]
	*In vivo*		Gelfoam gelatin sponges	143B Tk^−^ OS cell line	hMSCs	Bone	[46]
			PCL	SAOS-2 OS cell line	Osteoprogenito cells (bone compartment), hMSCs and HUVECs (vascular compartment)		[47, 48]

^*1*^*OS osteosarcoma, *^*2*^*ES Ewing sarcoma*

An easy way to mimic the bone marrow microenvironment was used by Han et al. to investigate the roles of chemokine receptors CXCR7 and CXCR4 in osteosarcoma invasion [33]. In their model, bone-marrow-derived mesenchymal stem cells (BMSCs) were co-cultured with human osteosarcoma cell lines MG-63 and U2OS in an indirect 2D transwell system. They investigated CXCR4, as its suppression has been shown to reduce osteosarcoma cell invasion and metastasis [49]. However, successful suppression of CXCR4 alone did not affect invasion in the co-culture model, as upregulation of CXCR7 due to CXCL12 secretion by the BMSCs lead to sustained invasive potential. Neither CXCR7 transfection of the osteosarcoma cell lines nor supplementing the growth medium with CXCL12 led to sustained cell invasion in the osteosarcoma cell lines alone. Thus, the study revealed that an unidentified factor of the BMSC microenvironment was necessary to observe the successful switch from CXCR4- to CXCR7-driven invasion, thereby highlighting the need to consider the bone microenvironment when looking for new drug targets. This study shows how even a relatively minor upgrade of a simple 2D model to a technically still feasible co-culture model can improve its relevance to a state more closely related to the patient. The model’s utility for drug testing is enhanced compared to the experiment using osteosarcoma cell lines only.

However, many studies have shown that 2D cell culture lacks important characteristics of 3D tissues and often overestimates response to novel therapies while underestimating chemoresistance [35, 36, 39, 50]. 3D culture systems are well known to be more capable of faithfully modelling osteosarcoma and its complex microenvironment and therefore seem to be more valuable for drug testing studies compared to 2D models. De Luca and colleagues gave an overview of 3D *in vitro* culture models to study the osteosarcoma environment [51]. They distributed the 3D model systems into scaffold-free and scaffold-based approaches:

Scaffold-free approaches are usually based on the formation of tumour spheroids. This can be achieved by the liquid-overlay technique, in which the cells are grown on non-adhesive surfaces like agar-agarose, poly-HEMA or low-binding plates and therefore form spontaneous aggregates [52]. Alternatively, spheroids can be generated through the hanging drop technique, where cell suspension droplets are applied to the lid of culture dishes and aggregate under the influence of gravity [51, 53].

Osteosarcoma spheroids can produce matrix [54]. However, these models are lacking the complex microenvironment of bone. To circumvent this, some authors co-culture tumour spheroids with other cell types of the bone niche, such as endothelial cells. For example, Chaddad and colleagues co-cultured osteosarcoma spheroids with human umbilical vein endothelial (HUVEC) cells grown on a 2D layer to mimic a vascular component [38]. Comparable to the previously discussed work by Han and colleagues, a 3D model system too can be made more relevant by introducing a second cell type enriching the tumour microenvironment and thus making the model potentially more relevant for preclinical drug testing.

Another method to create complex 3D *in vitro* platforms is by generating organoids. In the literature, the terms organoids and spheroids are sometimes used interchangeably. However, whereas spheroids usually self-assemble into simple cell clusters, organoids are typically made of progenitor cells that assemble with the aid of extracellular matrix structures and grow into more complex micro-organs. Tumour organoids were shown to keep the histologic and genetic features of the original tumour source [55]. This ability to mimic the disease more closely to the patient’s state compared to other *in vitro* model approaches, while at the same time being technically still feasible for laboratories, makes organoids of particular interest for drug testing and screening experiments. However, most work on tumour organoid models in the past was addressing tumours of epithelial cell origin. Tumour organoid models based on mesenchymal tissues are still relatively rare, and therefore, there are not many descriptions of osteosarcoma organoid models in the current literature [56].

As recently as 2022, Nie and colleagues presented their approach to establishing patient-derived organoids (PDO) from a total of 24 osteosarcoma patients [57]. PDOs were created by the digestion of fresh osteosarcoma samples into a single-cell suspension and mixed with Matrigel to provide an extracellular matrix structure. The study was conducted to investigate the frequency of glypican-3 (GP3) mutations in osteosarcoma as well as its potential as a therapy target. Even though GP3 mutations were not found in the investigated osteosarcoma samples and therefore understood as being rare occasions, antibody-targeted therapy for high GP3-expressing PDOs was successful.

The method used to generate PDOs in the work described above was based on an earlier paper by Aina He and colleagues describing the first patient-derived lung metastatic osteosarcoma organoid model [56]. Two different methods were used to create the PDOs: In addition to the already introduced single-cell method above, organoids were established based on structurally intact tumour pieces embedded in gel. More specifically, for this “Cut/EnBloc” method, surgically removed lung metastatic osteosarcoma tissue was minced into small pieces, embedded into a collagen-based gel without further digestion and placed on top of a second collagen gel layer in a transwell insert. The created PDOs were shown to maintain their histological characteristics and also T cells as part of the original immune niche. However, the continued presence of T cells required a unique supporting medium. The organoid itself could not retain the T cell population on its own.

In addition to organoids being able to recapitulate important characteristics of osteosarcoma and some microenvironment factors as valuable tools in drug testing, the presented models importantly use fresh patient-derived tissue instead of established cell lines. We believe using native patient tissue is beneficial as the resulting model will better represent the patient’s disease specifics compared to a cell line. Those PDOs can be used to create a panel of personalized test platforms, opening up the possibility to check for and test new drugs for particular subgroups of osteosarcoma patients. For example, as Nie and colleagues did by focusing on GP3 mutations. Furthermore, we find the “Cut/EnBloc” method of particular interest, as existing cell-to-cell and cell-to-matrix connections remain intact. However, whether this method is ultimately superior compared to the generation of organoids using single-cell suspensions is unknown. A challenge of PDOs is access to patient material, which is limited and often times difficult to arrange, and therefore might be a barrier for many labs.

Like the organoid models presented above, scaffold-based approaches generally provide a given 3D structure. In the case of some natural scaffolds, cancer cells grow on and interact with the extracellular components. Commonly used natural scaffolds include alginate, Matrigel, collagen, chitosan, silk and methylcellulose matrices [51, 53].

In addition to natural scaffolds, the use of synthetic scaffolds, such as poly(ε-caprolactone) (PCL) and poly(lactid-co-glycolid) (PLGA), shows increased popularity in modeling the bone environment. Those scaffolds are cost-effective and allow for easy manipulation that caters to the individual research goal [58].

**Fig. 2:**
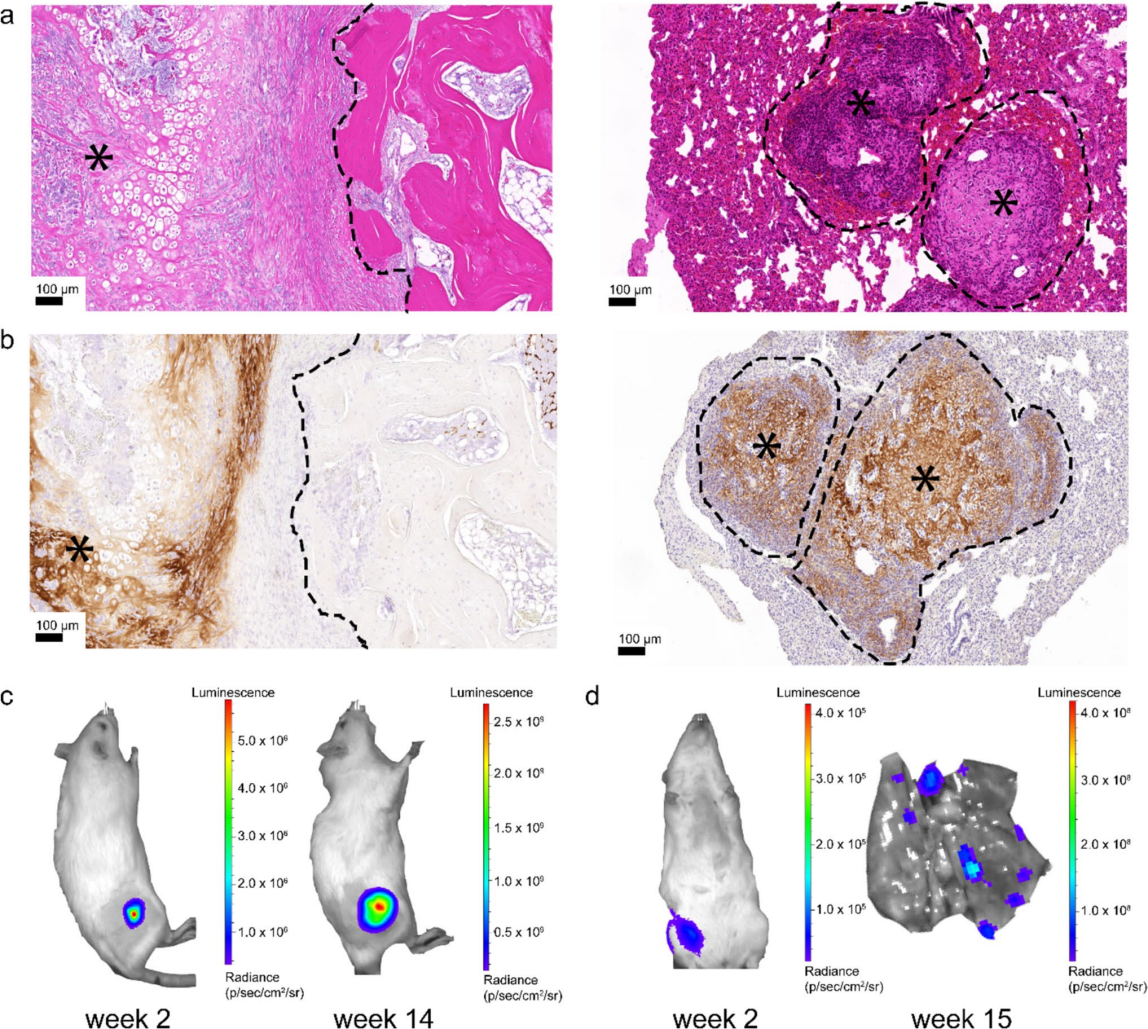
A humanized bone niche serves as a platform for human osteosarcoma development and metastasis. **a** H&E images of the bone niche (black dotted outline) and primary osteosarcoma tumour generation from an intraosseous injection of the human SaOS-2-luc cell line (left image). Spontaneous metastases developed in the rat lungs over a 15-week period (right image). **b** IHC for human-specific collagen type 1 confirms the presence of human ECM created by the primary osteosarcoma tumour (left image) and demonstrates the development of a supportive human-derived ECM matrix within the developing lung metastases (right image). **c** *In vivo* bioluminescent imaging demonstrates the growth of the SaOS-2-luc primary tumour over time, **d** as well as the development of distant metastases that were not detectable 2 weeks after primary tumour establishment, but were detectable at the endpoint as confirmed by ex vivo BLI

To mimic bone-disease interactions in preclinical tumour models as well as to overcome interspecies differences *in vivo*, the Hutmacher group established a sophisticated humanized bone organ model, further referred to as humanized tissue-engineered bone construct (hTEBC) [59, 60]. The bone organ model consists of calcium phosphate-coated 3D-printed medical-grade PCL scaffolds seeded with human osteoprogenitor cells [59–61]. Additionally, in more recent studies, a vascularized bone marrow niche was incorporated, consisting of HUVECs and mesenchymal stem cells (MSCs) embedded in gelatin-methacryloyl (GelMA) hydrogel [62–66]. The hTEBCs are a humanized bone niche originally established in immunodeficient NSG mice. Their application in *in vivo* studies during the last decade showed that the hTEBCs can faithfully mimic some aspects of human bone. They contain human bone cells, human-derived extracellular matrix, trabeculae, bone marrow compartment and complex remodelling through endochondral ossification (see Fig. 2a and b, left side) [59, 67]. An orthotopic model using hTEBCs implanted around the femur of NSG mice was created to study primary bone malignancies [47] and was established in X-SCID and more recently by the McGovern lab in Il2rg, Rag2 double knockout rats to allow for resection surgery experiments (Fig. 2) [48]. The osteosarcoma cell line, SAOS-2, was injected into the bone niche. It formed spontaneous lung metastasis of human origin in the *in vivo* model (Fig. 2a and b, right side), which are calcified and present in CT imaging (Fig. 2d). Compared to the other models introduced so far which attempt to model some aspect of the tumour environment, the hTEBC model aims to mimic healthy human bone more holistically. Being able to mimic the bone environment more completely compared to other bone microenvironmental models might provide an advantage in drug testing. However, the more complex model also requires more significant resources, including the know-how to produce the 3D-printed scaffolds (or access to buy them).

Furthermore, setting up the model is very time-consuming with a minimum of 2–3 months from assembly of the hTEBCs until use in an experiment. Using the hTEBCs in a mouse model has the advantage of creating a humanized bone niche in an animal whose bone structure has otherwise distinct differences from human bone. By creating the humanized bone mouse model, species differences are being minimized and therefore drug efficacy studies are likely to be more relevant to patients. Another advantage is the mouse models ability to metastasize, which is not possible to assess in *in vitro* models and could be particularly interesting for testing drugs for progressive disease states. As effort and expenses of studies using the hTEBC *in vivo* model are high, the model is less interesting for drug screening purposes but beneficial for testing drugs that were already determined as potentially potent in *in vitro* studies.

PCL is a popular material for scaffold-based 3D models. It is used by other groups as well: the Mikos research group for example generated PCL-scaffold based *in vitro* models for Ewing sarcoma. While the Hutmacher group aimed to create a fully tissue-engineered bone niche, the goal of the Mikos group is to model and investigate the acellularized bone ECM in particular. Hence, they seeded human mesenchymal stem cells onto 3D-printed PCL scaffolds and cultivated them by adding osteogenic supplements. Afterwards, the scaffolds were decellularized, whereas the produced extracellular matrix remained and provided bone ECM for the subsequently added TC-71 Ewing sarcoma cell line [41].

In this section, we provided a concise overview of current techniques for investigating osteosarcoma in the context of its microenvironment. Even partially modelling the osteosarcoma microenvironment is tremendously complex, as evidenced by the highly diverse model systems developed by various research groups (as summarized in Table 1). Each model system presented has its strengths and weaknesses regarding their utility for drug testing and none can faithfully recapitulate the entirety of a patient’s bone microenvironment. Some authors question the reliability of model systems that cannot recapitulate the whole human microenvironment [19, 53]. We postulate this is a reasonable doubt and warrants careful consideration when interpreting results, particularly in the context of clinical translation. Although the complement of bone microenvironmental factors relevant to osteosarcoma is unknown, the partial models discussed above still serve as valuable tools to advance our understanding and serve as drug testing platforms. We expect the field of microenvironment models for osteosarcoma will continue to expand and refine further within the coming years, with a focus on incorporating additional microenvironment factors and developing more sophisticated models.

Most studies described in this chapter, from osteosarcoma spheroid-based studies to more complex tissue-engineered bone models, use established cell lines (see Table 1, tumour cell source). Although human-origin cells are primarily used, canine and murine cells have also been frequently described. In DeLucas’ 2018 review, only a single paper is cited using osteosarcoma cells isolated from fresh human samples for a spheroid study [51, 68]. Established cell lines are inexpensive, readily sourced and easy to handle, so we believe they are an essential and powerful tool for understanding disease processes and validating new models. However, due to evidence of cell lines veering from the original patient’s disease over time [4, 69] as well as the emerging importance of accounting for patient-variability, fresh patient-derived cell and tissue sources are of critical importance moving forward [5].

### Patient-derived xenograft mouse models

Patient-derived xenograft (PDX) mouse models are considered the current gold standard of cancer modelling and are already used in osteosarcoma research [70, 71]. The term “PDX model” serves as an umbrella term referring to the implantation of a surgically removed patient-derived tumour into an immunodeficient mouse [72]. The PDX tumours were shown to display very high similarity compared to the original patient’s tumour at a histological, genetic and epigenetic level [73, 74], making them of particular interest for drug efficacy studies. Furthermore, the tumour tissue contains some host stroma and extracellular matrix architecture, even though mouse stroma and vasculature are shown to infiltrate rapidly [75].

#### Variety of PDX models

However, the methodological details used to create “PDX models” may differ widely between different research groups: Generally, after the initial implantation, the tumour tissue is allowed to grow *in vivo* until a defined size and then explanted, cut into smaller pieces and reimplanted into a new mouse host, until there is enough for an experiment. This “passaging” of the tumour tissue from one mouse through another to expand the patient tissue is comparable to the expansion of cells in cell culture [72, 76].

PDX models vary in pre-implantation tissue processing techniques and implantation site, as summarized in Table 2. The most straightforward method is the subcutaneous (s.c.) implantation of unprocessed patient-derived tumour tissue. For example, Nanni et al. generated PDX models by implanting fresh osteosarcoma and Ewing sarcoma tissue s.c. at the level of trans-scapular brown fat of male NSG mice. They observed great genetic and morphological similarities between the initial patient-tumour and the PDX models. Additionally, they isolated patient-derived cell lines from patient samples. However, the patient-derived cell lines were inferior in similarity to the patient tumour compared to the PDX tissue [77]. In another example, Zhou et al. implanted patient osteosarcoma tissue s.c. into BALB/c nude mice to investigate the role of the E3 ubiquitin ligase TRIM7 on osteosarcoma tumorigenesis and chemoresistance [78]. They divided the PDX models into two groups, with low or high TRIM7 expression, and compared the efficacy of adriamycin and methotrexate chemotherapeutics in reducing the tumour burden. Although chemotherapy resulted in a statistically significant reduction of all tumours, the tumours of the high-TRIM7 group remained larger compared to the low-TRIM7 expressing group. Thus, the authors concluded that TRIM7 might play a role in chemoresistance.

**Table 2:** Osteosarcoma PDX models: overview of commonly used techniques

Implantation type	Implantation site	Mouse strain	Literature
Subcutaneous	Flank	NSG NOD-SCID SCID Athymic nu/nu C.B-lgh-1b/lcrTac-Prkdcscid	[79] [80] [73] [80–82] [83]
	Trans-scapular brown fat	NSG RGKO	[77]
	Not specified	NSG BALB/c-nu/nu	[84] [78]
Orthotopic	Femur	Athymic nu/nu	[85, 86]
	Tibia	Athymic nu/nu Athymic BALB/c nu/nu	[87] [88]
Other	Subrenal capsule	NSG	[24]

To further advance the similarities between the PDX approach and the situation in the patient, PDX models were established where the tumour tissue is implanted at an orthotopic site, most commonly the mouse femur or tibia. Blattmann and colleagues were the first to publish their approach of an orthotopic osteosarcoma PDX model using fresh patient material in 2015 [88]. In their approach, they drilled a 0.5 mm hole in the central part of the right tibia of athymic BALB/c nude mice and placed a 1 × 1 × 1 mm^3^ tumour piece in contact with bone marrow within the cavity. They reported that 50% of the PDX mice developed a visible tumour mass after 40 days. However, the time until the tumour could be detected was shortened with every further passage. Interestingly, they also isolated a primary osteosarcoma cell line by alternately passaging the cells in cell culture and mice. By doing so, Blattmann and colleagues achieved high genetic and histological similarity of the PDX model and the primary cell line compared to the patient tumours. In addition to their orthotopic PDX model, s.c. PDX models were established. Genetic analysis showed differences compared to the genetic profile of the patient-tumour that were not evident in the orthotopic model, possibly indicating the superiority of the orthotopic approach. However, the genetic differences in the s.c. model were not believed to be driver mutations for OS.

Su and colleagues used a patient-derived orthotopic xenograft mouse model for osteosarcoma by implanting a tumour fragment into the mouse femur [85]. More precisely, they resected the lateral condyle of the femur of athymic Nu/Nu nude mice and placed a fresh 3–4 mm tumour fragment into the created hole. In the study, the PDX was used to investigate a novel CDK-9 inhibitor, compound 5k, which was shown to reduce tumour growth significantly.

The research group around Robert M Hoffman established various PDX models, including multiple models for primary bone tumours such as osteosarcoma, to test novel drug combinations. Besides the s.c. PDX model [81] and orthotopic tibia [87] and femur models [86], they developed a model for OS lung metastases [89]. Osteosarcoma lung metastase tissue, previously expanded in another PDX model, was sewn into the lower lungs of athymic nu/nu nude mice.

Besides the most common osteosarcoma models presented above, PDX models can be immensely useful in creating sustainable models for particularly rare osteosarcoma subcategories. Therefore, models of primary breast and jaw osteosarcoma have been established and provide a valuable resource for studying their unique tumour biology in the future [90, 91].

#### Minimal information standards for PDX models

Altogether, a wide range of osteosarcoma PDX models currently exist. Those PDX models differ widely in their establishment and validation methods and quality assurance procedures to verify continued similarity to the initial patient tumour. In an attempt to introduce a standard set of rules used amongst the PDX community, multiple authors proposed guideline papers on how to establish and document PDX models adequately. Meehan et al. proposed “PDX-Minimal Information” standards, differentiating essential and desirable information to be collected about the patient, their clinical history related to the tumour, model creation, quality control and research studies conducted with the PDX [92]. They based their PDX-MI standard on already available standards developed by the EurOPDX consortium [93], the IMODI consortium, the Patient-Derived Models Repository at NCI-Frederick and The Jackson Laboratory PDX Resource [94]. Stripecke et al. recently proposed a checklist for “Minimal Information for Standardization of Humanized Mice”, including PDX models. [95] Additionally, authors like Mattar and colleagues provide in-depth guidance on establishing and maintaining PDX models, including how to properly perform quality assurance [96]. The Mattar paper also impressively highlights the extensive team of specialists, time and resources required to successfully establish PDX models.

#### PDX models for personalized medicine

The establishment of osteosarcoma PDX models is very time-consuming, and PDX models are only partially fit for personalized medicine approaches. In their study, Su and colleagues determined that 50% of included patients could have theoretically benefited from results obtained from their PDX models [85]. In an attempt to increase the usefulness of PDX models for personalized medicine, researchers like Sayles and colleagues proposed a “genome-informed” approach to personalized osteosarcoma treatment [24]. In their paper, they performed extensive genomic analysis of the patient tissue used to create PDX models. Based on the tumour-specific mutations, treatment strategies were proposed, tested and compared to the tumours with different mutations. They found that they could successfully match different treatment approaches according to the obtained genetic data and that no tested drug was equally beneficial for treatment in all of their PDX models. This study highlights how a single disease model system is not universally applicable for all types of osteosarcoma and a panel of patient-specific disease models for osteosarcoma is highly desirable.

Similarly, the US Paediatric Preclinical Testing Consortium recently published extensive genomic datasets of 261 PDX models of 29 different paediatric cancers, including 36 osteosarcoma and 10 Ewing sarcoma models [83]. All models and genomic datasets are available to the scientific public, and the authors hope their data fosters a rational genome-matched clinical design for rare paediatric tumours.

For further information about osteosarcoma PDX models, specifically in the context of genome-driven therapy evaluation, the recent review of Landuzzi and colleagues provides a complete overview [21].

#### Challenges in creating and utilizing PDX models

As outlined above, PDX models are complex disease models that come with a high workload and cost that require a whole team of experts for successful high-quality establishment and maintenance [96]. Besides the great potential of those models to foster translatability of experimental drug testing results into the clinic and to better understand particularly rare tumour entities as well as explore more personalized treatment approaches, there are some shortcomings and difficulties of the method that will be addressed below.

A key step in establishing a PDX model is the successful engraftment of the tumour tissue in the mouse. Engraftment rates reported for osteosarcoma PDX models vary widely, ranging from around 40 [77, 80, 97] to almost 80% [82] in recent publications. Different factors, including the mouse model, time until implantation of the tumour tissue into the mouse host and tumour-specific factors, are believed to influence engraftment rates.

Tumour xenograft tissue cultivation requires the use of highly immunodeficient mouse strains, like the athymic Nu/Nu mouse strain, SCID or NSG mice. These strains have superior engraftment rates in severely immunocompromised mice like NSGs [98]. However, as highly immunocompromised mouse strains are generally more expensive, some groups prefer to switch the mouse strain after successful first engraftment [80].

Another important factor influencing successful implantation is the time between the excision of the fresh tumour tissue from the patient until implantation into the mouse. The optimal time limit was between 30 min and 1 h. However, staying within that very short time limit may not be feasible (e.g. due to infrastructural difficulties: collecting the sample from the operating room, transport to the pathology for evaluation, and transport to the animal facility). Thus, keeping the fresh tumour samples in preservation media like Hypothermosol™ until implantation is possible may enhance engraftment by prolonging tissue vitality [96, 99].

Furthermore, the tumour tissue itself heavily influences the chances of engraftment. Tissue from advanced disease appears to be more likely to result in PDX engraftment, as reported by Fortuna-Costa, who found higher success rates in samples of patients with metastatic disease at presentation [97]. This observation is not exclusive to osteosarcoma PDX models, as it was also reported that more aggressive forms of breast cancer show higher engraftment rates [100]. Moreover, Castillo-Ecija and colleagues found that PDX engraftment can predict the aggressiveness of the disease of paediatric osteosarcoma, Ewing sarcoma and rhabdomyosarcoma patients [80]. They also observed that PDX engraftment is more likely to occur in more aggressive tumours and therefore found that PDX engraftment can be used as a prognostic factor for newly diagnosed patients determining poor outcomes including reduced life expectancy.

Matching this data, Nanni and colleagues reported a 100% engraftment rate of extraskeletal OS tissue (compared to 30% of bone OS) which is known to have a poorer overall prognosis [77]. Moreover, they found that engraftment rates were specimen-specific rather than patient-specific: Only in one out of five cases in which they could obtain tissue from two tumour sites of the same patient, both tissues could be successfully engrafted into the mouse model. Additionally, after implantation, tumour growth rates varied widely from 1 week to 1 year until a sizeable tumour appeared.

The treatment history of a patient may also affect engraftment rates, as neo-adjuvant chemotherapy lowered the chance of engraftment for osteosarcoma samples as reported by Nanni [77]. On the other hand, Fortuna-Costa and colleagues observed higher engraftment rates of post-chemotherapy samples [97].

Besides potential difficulties facing the successful engraftment of tumour tissue, other organisational pitfalls may arise. Access to tumour samples can be difficult for research facilities for multiple reasons. First, osteosarcoma patient tissue is generally limited by the rarity of the disease. Another limiting factor is the tumour sample size. Especially in small biopsy specimens or tumours with large necrotic areas, viable tissue volume is low [82]. Furthermore, many research laboratories are not connected to a (university) clinic, and as such face barriers in establishing hospital contacts and potentially more complex organisation is required to obtain tumour samples. Generally, as already outlined above, an interdisciplinary team of surgeons, pathologists, researchers and support staff working in all involved departments from the operating room to the laboratory are needed to establish and maintain PDX models successfully.

#### Shortcomings of the method

Even though PDX models recapitulate the patient’s disease very closely, there still are genetic and epigenetic changes compared to the original state of the tissue [73, 74]. It is also important to keep in mind that spontaneous murine tumours may arise in PDX models [101]. Thus, regular quality control checks are essential. Thinking about treatment studies beyond chemotherapeutics, using immunodeficient mouse strains makes trials with immunotherapies difficult, as a minimal host immune system is available [102]. Perhaps, further humanization of the PDX model by introducing a human immune niche would solve that problem as done successfully in the past [71, 103, 104]. However, this adds another complex step to an already time-consuming, expensive, complex model.

Another critical aspect of osteosarcoma that a mouse cannot fully recapitulate is the human bone environment and its interaction with the tumour, due to interspecies differences. Even though the implanted tumour tissue contains some host stroma and extracellular matrix architecture, the human tissue may be infiltrated and even overcome by mouse stroma [105].

Importantly, metastasis—especially to the lung—is a crucial aspect of osteosarcoma that seems challenging in some PDX models. The ability of PDX models to recapitulate spontaneous metastases *in vivo* is controversial in the literature [106, 107]. It is reported that spontaneous metastases occur more likely in orthotopic than in subcutaneous PDX models [72, 93, 107]. However, none of the PDX models cited in this chapter were reported to create spontaneous lung metastasis.

In summary, PDX models are currently the closest to the actual patient disease and provide a tremendously important platform expected to advance osteosarcoma treatment in the following years. The quality of the model system, however, comes at a high cost of resources and time, as well as faces real challenges regarding planning and organising that may be too high for some laboratories. Thus, more extensive drug screening experiments might be more feasible in less expensive and elaborate preclinical models like organoids, which still show remarkable similarity to the patients’ disease. However, PDX models are very relevant for further validating the efficacy of drugs deemed interesting in previous screening experiments and a valuable tool for increasing available tissue sources. Regrettably, especially human microenvironment-tumour interactions cannot be fully addressed in those mouse models due to a lack of human(ised) bone niches and a fully functional (human) immune system. Difficulties in forming spontaneous metastasis is also a problem, as treatment of lung metastases is considered one of the most important current challenges in osteosarcoma research.

### Patient-derived *in vitro* models

There is a great body of literature describing *in vitro* osteosarcoma studies, usually using well-established osteosarcoma cell lines [51, 108]. However, the existence of freshly patient-derived *in vitro* models for osteosarcoma, which we believe to be more useful tools for investigating new drugs for osteosarcoma, is still comparatively sparse in the literature. The recently described patient-derived osteosarcoma organoid models, already discussed in Sect. 3.1, are great exceptions to this general observation. The generation of PDO and PDX models perhaps indicates that change is happening. Moreover, oftentimes patient-derived cells (PDCs) are isolated from or during the creation of *in vivo* PDX models, as discussed previously in more depth [77, 88]. Those PDCs are used to confirm research results obtained with established cell lines [109] and extend the pool of available cell sources to receive more relevant outcomes in the *in vitro* experiments [110, 111]. Moreover, as PDX models are very time-consuming and expensive, the use of PDCs in the *in vitro* setting allows for a more cost-effective early screening of potential new drugs and methods [112]. As more PDX models and corresponding PDC lines are generated and become more accessible, we expect the use of PDCs to become even more important in osteosarcoma research in the following years.

### Alternative animal models for osteosarcoma research

While mouse models probably are the most widely used as well as a cost-effective animal model for various diseases including cancer, alternative animal models may provide additional advantages. For example, rats are more suitable when more complex surgical procedures are planned due to their bigger size. Thereby, drug studies exploring targeted treatment approaches, for example by surgically placing drug-loaded carriers close to the site of the disease—in the context of a biopsy or after resection—, are more practicable in rats compared to mice. At the same time, the still relatively small size and subsequent low costs in housing are attractive for laboratory use [48]. Furthermore, large animal models such as dogs and pigs are used as well. Both species show great similarities to human physiology as well as bone biomechanical properties that are unmet in rodents [113]. Moreover, large pet dogs often develop osteosarcoma naturally, which is histologically undistinguishable from human disease. Hence, veterinary studies of canine osteosarcoma may be of value in understanding the human disease and vice versa [114]. On the downside, strong ethical concerns around dogs, especially used as controlled animal models in scientific research facilities, exist due to their popularity as pets. This, however, is not the case for pigs, as they are widely accepted as a food source [115]. The biological similarities between pigs and humans as well as few ethical concerns make them an attractive animal model. Furthermore, their lifespan of up to 10 years makes studying recurrent cancer, second-line treatments as well as the long-term effects of therapies possible. As minipigs are approximately the size and weight of humans, they can be imaged with standard patient imaging systems and therefore used to advance imaging regimens [116]. On the downside, animal husbandry is much more time and cost-intensive compared to rodents [117], making them less relevant for drug screening or early efficiency testing.

In the following, we want to briefly introduce current rat and pig models for osteosarcoma.

#### Rats

There are limited studies which have successfully modelled osteosarcoma in rats. Disease modelling *in vivo* is hindered by the lack of consensus on osteosarcoma genetic origin, which prevents the generation of spontaneous osteosarcoma *in vivo* model. One of the few spontaneous rat osteosarcoma models was developed by Hansen et al., through the introduction of a p53 knockout allele in a Fischer-344 rat [118]. The rat model developed spontaneous osteosarcoma in the long bones and developed pulmonary metastases. However, the rats also developed meningeal sarcoma with high frequency and thus was not a specific osteosarcoma model. The utility of this rat model for drug testing is therefore limited in our opinion due to the unreliable occurrence of the target disease as well as possibly not being able to recapitulate the underlying genetic complexity as prevalent in human patients.

In addition, the inherent difficulties in modifying the rat genome as compared to the mouse genome, has until recently resulted in a paucity of the availability of immunocompromised rat models. Cherrier et al. (2005) created one of the first spontaneous metastasis osteosarcoma models in immunocompromised rats [119]. The authors injected a high density of the OSR rat osteosarcoma cell line into the femur of Cyclosporin A-treated Sprague Dawley (SD) rats and observed a comprehensive primary tumour and distant pulmonary metastases over a 9-week period. In another orthotopic study which developed lung metastases, the UMR-106 rat OS cell line was intrafemorally injected into Cyclosporin-treated SD rats. The osteosarcoma model was used to study the efficacy of boron-mediated boron neutron capture therapy [120]. While we find those osteosarcoma rat models more useful and versatile for drug tests compared to the previously discussed p53 knockout model, the fact this is a non-humanized rat model might still be a barrier when translating any drug testing results to humans due to interspecies differences.

Recently, highly immunocompromised rats based on Il2rg knockout models have been developed [121, 122]. This rat model has allowed for the generation of human osteosarcoma xenografts which recapitulate the human disease processes (described above in section 3.1 see Fig. 2), and therefore may be a more relevant model to use for drug tests. [Gospos et al., unpublished data].

#### Pigs

Similarly to the rat models, osteosarcoma pig models are rare and the few genetically engineered models are not necessarily specific for osteosarcoma. Sieren et al. created a p53 mutant model in Yucatan miniature pigs by introducing a missense mutation into the *TP53* gene. The mutant pigs in the study developed not only osteosarcoma but also lymphomas and in one case nephroblastoma [123]. Interestingly, the Schnieke group was able to establish a p53 knockout Landrace pig model in which the occurrence of osteosarcomas as the only tumour type was observed [115, 124].

Furthermore, several research teams are working towards generating severe immunodeficient pigs. One example was developed by Itoh et al.: immunodeficient pigs were obtained by surgical removal of the thymus and spleen and subsequent drug immunosuppression [125]. Moreover, several immunodeficient pig models were created by disruption of the IL2RG and RAG2 genes or generation of double knockouts [117]. The generation of severe immunodeficient pigs allows for future humanized pig studies and the creation of pig patient-derived xenograft tumour models [116, 117], which might become very interesting for advanced and second-line drug tests as well as studying complementary disease management in the future.

## Conclusion

Besides tremendous research efforts to better understand osteosarcoma and to find new therapies, the translation of new treatment options into the clinic was largely unsuccessful during the last few decades. As the use of insufficiently accurate preclinical models is believed to be a key reason for the lack of progress, model systems that more closely resemble the disease state in the patients are continuously created and refined. Specifically, this includes the use of patient-derived tissues and the incorporation of a human bone environment, which are useful for drug testing in different stages. As presented in this review and summarized in Table 3, there have been extensive efforts to create and use complex models resembling the tumour and bone microenvironment more closely. Furthermore, PDX models provide a growing pool of tissue for drug efficiency studies that are as close to the original patient’s disease as currently possible. PSCs and PDOs derived from PDX models, moreover, provide easier and more cost-effective tools to address patient-specificity for drug screening and early drug efficiency studies and provide an increasingly available alternative to established osteosarcoma cell lines. The perspective of being able to generate humanized larger animal models in the future might also advance knowledge on second-line therapies and long-term treatment. We expect that new promising therapies will eventually make the jump into the clinic based on these new and more reliable models. Additionally, refinement and combination of currently available techniques will further improve the models and ultimately our understanding and treatment strategies of osteosarcoma.

**Table 3:**
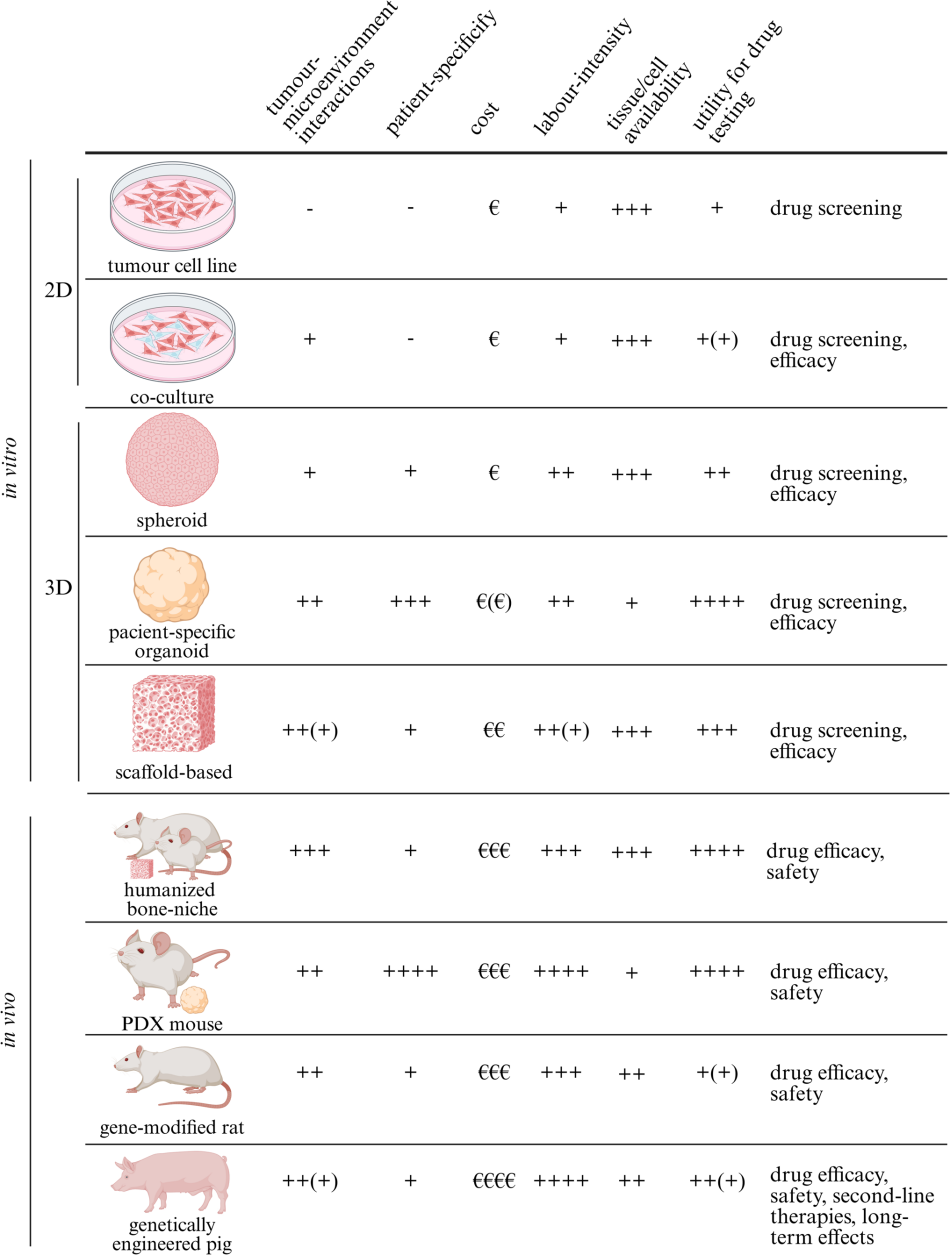
Characteristics of current osteosarcoma models and their utility in drug testing

## Data Availability

No datasets were generated or analysed during the current study.
